# The Compositional and Functional Attributes of Commercial Flours from Tropical Fruits (Breadfruit and Banana)

**DOI:** 10.3390/foods8110586

**Published:** 2019-11-19

**Authors:** Shiqi Huang, Mario M. Martinez, Benjamin M. Bohrer

**Affiliations:** 1 Department of Food Science, University of Guelph, Guelph, ON N1G 2W1, Canada; shuang11@uoguelph.ca; 2 School of Engineering, University of Guelph, Guelph, ON N1G 2W1, Canada; mario.martinez@uoguelph.ca

**Keywords:** flour, banana, breadfruit, composition, pasting properties, thermodynamics, retrogradation

## Abstract

The objective of this study was to compare the compositional and functional properties of tropical flour sources (two breadfruit flours (type A and type B) and a banana flour) with a more traditional flour source (wheat flour). Macro-nutrient composition, pH, water and oil holding capacity, bulk density, particle size, solubility, swelling power, pasting properties, and thermodynamics (gelatinization and retrogradation) were determined. All flours evaluated were similar in their composition with high levels of carbohydrates (greater than 82.52 g/100 g on a dry-matter basis), with most of the carbohydrate content comprised of starch (greater than 67.02 g/100 g). The tropical fruit flours had greater (*p* < 0.05) water holding capacity than wheat flour. Breadfruit flour B had the lowest (*p* < 0.05) bulk density, while banana flour had the greatest (*p* < 0.05) bulk density. The swelling power of the tropical flours was greater (*p* < 0.05) than the wheat flour. The viscosity of the tropical flours was higher than wheat flour but decreased significantly when temperature was held at 130 °C. These results indicated that the two breadfruit flours and banana flour have great potential for application in processed food products, and have similar compositional attributes to a more traditional flour.

## 1. Introduction

Starches are an important ingredient used for many purposes in the food manufacturing industry. Common sources of starches include those from some of the most commonly grown staple crops in the world like wheat [1,2]. There is a goal among food scientists to adequately characterize flour from unique staple crops for future use in processed food products. Tropical climates are suitable for production of many unique staple crops such as breadfruit [3] and banana [4]. Breadfruit is a high-yielding traditional staple crop found throughout Oceania, and one great advantage of this crop is that the tree does not need to be replanted each year [5] and its production lifespan is more than 50 years [6]. The crop harvested from the plant has high levels of carbohydrates, fiber, vitamins, and minerals [3]. Breadfruit is also a rich source of starch and has shown promise as a food ingredient when incorporated into meat products [7]. Bananas are grown in all tropical regions and constitute a major staple food crop in many economies in the developing world. Furthermore, bananas are the world’s fourth most valuable food crop in terms of gross production value behind rice, wheat, and corn [4]. The main source of carbohydrate in banana is starch [8]. It has been reported that consumption of a low glycemic index (GI) diet could decrease the possibility of type II diabetes onset for adults [9]. Turi et al. [10] concluded that cooked breadfruit has a low to moderate GI value. Banana was also categorized as a low GI value food [11]. Conversion of the fresh fruits to flour or isolated starch makes an ingredient that is in a more stable form and increases its versatility for use in processed food products [7,12,13,14]. Breadfruit flour and banana flour are both unexplored ingredients with great potential for application in many food products. Hence, a comparative characterization of their composition and functionality would provide valuable information to the food industry and advance the potential of these novel starchy flours to be used in processed food products. Therefore, the objectives of this study were to determine compositional and functional attributes (including macro-nutrient composition, pH, water and oil holding capacity, bulk density, particle size, solubility, swelling power, pasting properties, and thermodynamics) of two commercial breadfruit flours (sourced from Samoa and Costa Rica) and a commercial banana flour (sourced from the USA), and then form a comparison with a more traditional flour source like wheat flour.

## 2. Materials and Methods

### 2.1. Composition Analysis

Tropical flour sources evaluated in this research included two types of breadfruit flour and one type of banana flour. The first type of breadfruit flour (Breadfruit flour A) was provided by Natural Foods International (Apia, Western Samoa). The second type of breadfruit flour (Breadfruit flour B) was provided by Jungle Project/Tropico Agroforestal S.R.L. (Alajuela, Costa Rica). Both breadfruit flours were of the *Artocarpus altilis* origin. The banana flour was provided by LiveKuna and was manufactured by Kunachia LLC (Davie, FL, USA). The banana flour was of the *Musa acuminate* origin. Wheat flour was obtained commercially from a local ingredient supplier Bulk Barn Foods, Aurora, ON, Canada) located in Guelph (ON, Canada). The flours were used in their native state. Moisture, lipid, protein, and ash content were determined according to the AOAC methods 925.10, 920.85, 992.15, 923.03, respectively [15,16,17,18]. Total starch content was determined by AOAC method 996.11 with modification in accordance with the total starch assay kit instructions (Megazyme International Ltd., Wicklow, Ireland) [19]. Freeze-dried defatted samples were used for this test. Considering the flours might have D-glucose and resistant starch [10,20], the samples were washed with aqueous ethanol (80% *v*/*v*) to remove the D-glucose and stirred with 2M KOH at approximate 4 °C (ice/water bath) to pre-dissolve the resistant starch. 

Carbohydrate content was calculated according to an equation provided by the Food and Agriculture Organization of the United Nations [21] with minor modifications:

Carbohydrate (g/100 g) = 100 − protein content (g/100 g) − lipid content (g/100 g) − moisture content (g/100 g) − ash content (g/100 g),(1)

Calorie and energy content were calculated according to the following equation [22]:

Calories (kcal/100 g) = [4 × protein content (g/100 g)] + [4 × carbohydrate content (g/100 g)] + [9 × lipid content (g/100 g)],(2)

Energy (kJ/100 g) = 4.184 × Calories (kcal/100 g),(3)

### 2.2. pH

Ten g flour samples (in triplicate) were homogenized in 50 mL of distilled water for the determination of pH. pH values were quantified using a benchtop pH meter (AR15 Accumet Research, Thermo Fisher Scientific, Mississauga, ON, Canada) following calibration with buffer solutions of pH 4.0 and pH 7.0.

### 2.3. Water and Oil Holding Capacity 

Fifteen mL of distilled water (for the oil holding capacity determination, 15 mL of refined corn oil) was added to 1 g of the flour sample in a weighed centrifuge tube. The contents were mixed using a vortex mixer for 2 min, and then centrifuged for 20 min at 6000× *g* on a bench centrifuge (Model 21000, IEC International Equipment Company, Needham Heights, MA, USA). The clear supernatant was carefully removed and discarded. Water holding capacity was expressed as the weight (in g) of water bound by 1 g of dried flour sample. Oil holding capacity was expressed as the weight (in g) of oil bound to 1 g of dried flour sample.

### 2.4. Bulk Density

Fifty g of flour samples was placed in a 100 mL measuring cylinder. The cylinder was gently tapped on a laboratory bench several times to ensure a constant volume. Bulk density (g/cm^3^) was calculated as the weight of sample per unit volume of sample.

### 2.5. Particle Size Distribution

Particle size distribution of flour samples was measured using a Malvern Master Sizer laser diffraction analyzer (Mastersizer 2000, Malvern Instruments, Ltd.; Worcestershire, UK) at room temperature (approximately 21.5 °C) according to the methodology of Roman et al. [13] with modifications. The Mie theory was used with consideration of a refractive index of 1.53 and 1.33 for flour and dispersant (water), respectively. The particle size distribution of flour samples was recorded with reference to volume weighted mean diameter (D_4_,_3_). 

### 2.6. Differential Scanning Calorimetry (DSC)

The gelatinization and retrogradation process of the flours were characterized calorimetrically using a differential scanning calorimeter (DSC25; TA Instruments, Crawley, UK) equipped with a refrigerated cooling system (RCS 40; TA Instruments) and nitrogen purge gas. An amount of 6 ± 0.1 mg of each flour sample was accurately weighed and mixed with 18µL distilled water in T_zero_ hermetic aluminum DSC pans (TA Instruments). The sample pan was then hermetically sealed. The sealed samples were placed at room temperature (approximately 21.5 °C) for 24 h to equilibrate. An empty pan was used as reference. The first run was used to measure the gelatinization process: at a heating rate of 10°C/min over the temperature range from 25 °C to 100 °C. After the first heating run, the sample pans were stored at 4 °C for 7 days. After the 7-day period, the second run was conducted for the measurement of the retrogradation process: at a heating rate of 5 °C/min over the temperature range from 20 °C to 100 °C. Measurements were performed in three replications and results were presented as mean values. The onset temperature (T_o_), peak temperature (T_p_), conclusion temperature (T_c_), and enthalpy (ΔH) of gelatinization and retrogradation were obtained by integration of the endothermic peak from DSC thermograms.

### 2.7. Solubility and Swelling Power 

One wt% aqueous suspension of each flour sample was heated to pre-determined temperatures (30 °C, 50 °C, 70 °C, and 90 °C) for 1 h with constant stirring using a magnetic stir bar and a stirring hotplate (Fisherbrand Isotemp stirring hotplate; Thermo Fisher Scientific, Mississauga, ON, Canada). Samples were poured into a weighed centrifuge tube and centrifuged at 3000× *g* for 10 min. The supernatants were carefully poured into weighed aluminum dishes and evaporated at 100°C using a drying oven (Fisherbrand Isotemp 180 L drying oven; Thermo Fisher Scientific, Mississauga, ON, Canada) for 24 h. The weight of dry solids was determined for the calculation of solubility. The weight of wet sediments in the centrifuge tube was determined for the calculation of swelling power:

Solubility (%) = (the weight of flour dissolved in water/the weight of total flour sample) × 100%,(4)

Swelling power (g/g) = the weight of the wet sediments/[the weight of total flour sample × (100% − solubility)],(5)

### 2.8. Pasting Properties

Pasting properties of the flour suspension were analyzed by two different methods. The first method was conducted with a Rapid Visco Analyser (#RVA-4, Newport Scientific Inc., Hägersten, Sweden), where the maximum temperature was 95°C. It is noteworthy that some starchy flours, such as banana starch, has been reported to exhibit an underdeveloped pasting profile or, more specifically, a lack of breakdown and setback upon heating [15]. For this reason, a second method was conducted using a Rapid Visco Analyser 4800 (RVA 4800, Perten Instruments; a PerkinElmer Company, Macquarie Park, Australia), where the maximum temperature was 130 °C. The viscometer RVA 4800 was equipped with a well-designed canister that can be sealed and self-pressurized up to 100 psi [23]. The high-temperature capability of RVA 4800 was expected to fully enable the swelling of the starch granules contained in the compacted monocarp tissue matrix of the tropical fruits. In other words, this would provide fully developed pasting profiles and additional rheological indicators describing the behaviour of the tropical fruit flours used in this study. 

With both methods, the moisture of flour was first measured by a moisture meter (MB45, OHAUS Corp.; Parsippany, NJ, USA) to obtain the correct flour sample weight and amount of water required for the test. The flour suspension (28 g total weight for the first method and 28.5 g total weight for the second method) was poured into an aluminum sample cup and the cup, with a plastic stirring paddle, was inserted into the RVA machine. For the first method, the sample temperature was held at 50 °C for 1 min, increased to 95 °C, held at 95 °C for 2.5 min, lowered to 50 °C, and held at 50 °C for 2 min. For the second method, the sample temperature was held at 50 °C for 1.5 min, increased to 130 °C, held at 130 °C for 3 min, lowered to 50 °C, and held at 50 °C for 2 min.

### 2.9. Experimental Design and Statistical Analysis

Proximate composition of flours was conducted in duplicate for each flour type. All other analyses were conducted in three replications for each treatment (flour type). Statistical analyses were performed with SAS (SAS 9.4, SAS Inst. Inc., Cary, NC, USA). Data (bulk density, pH, water and oil holding capacity, particle size, pasting properties, gelatinization, and retrogradation properties) were analyzed with PROC GLIMMIX of SAS with fixed effects of flour type and a random effect of replication. Data for solubility and swelling power were analyzed with PROC GLIMMIX of SAS with fixed effects of flour type and temperature, and a random effect of replication. Least square means were separated using the PDIFF option with a Tukey-Kramer adjustment. Differences were considered statistically different at *p* ≤ 0.05. 

## 3. Results and Discussion

### 3.1. Proximate Composition

The main compositional component of the two breadfruit flours, banana flour, and wheat flour was carbohydrates (which ranged 82.52 to 91.68 g/100 g on a dry matter basis), with most of the carbohydrates comprised of starch (which ranged from 67.02 to 77.14 on a dry matter basis; Table 1). The tropical flour sources had similar levels of protein (which ranged from 3.91 to 4.99 g/100 g on a dry matter basis), while wheat flour had significantly greater protein content (14.51 g/100 g on a dry matter basis). All the flours had low fat content (ranging from 0.97 g/100 g to 2.50 g/100 g on a dry matter basis).

The different starch content of the two breadfruit flours was likely caused by different variety, maturity stage, and climatic/agronomic conditions. The high starch content should make these flours promising ingredients, and improve the technological properties of food products prepared with these flours when compared with other sources of flour. While, protein make flours nutritionally important when compared with isolated starches, high protein content in flours can affect surface charge and rate of hydration, thus affecting starch swelling and gelatinization during cooking [24,25]. Lipid content might also affect starch swelling due to the formation of helical inclusion complexes with amylose [25,26]. Both the protein and lipid composition should be considered when working with and characterizing flours.

### 3.2. Functional Properties

#### 3.2.1. pH, Water Holding Capacity, Oil Holding Capacity, Particle Size, and Bulk Density

pH of the flours ranged in value from 5.07 to 6.09, with banana flour having the lowest (*p* < 0.05) pH value and breadfruit flour A having greatest (*p* < 0.05) pH value (Table 2). Previous research has reported that flour solubility and emulsifying activity are affected by pH [27].

**Figure 1:**
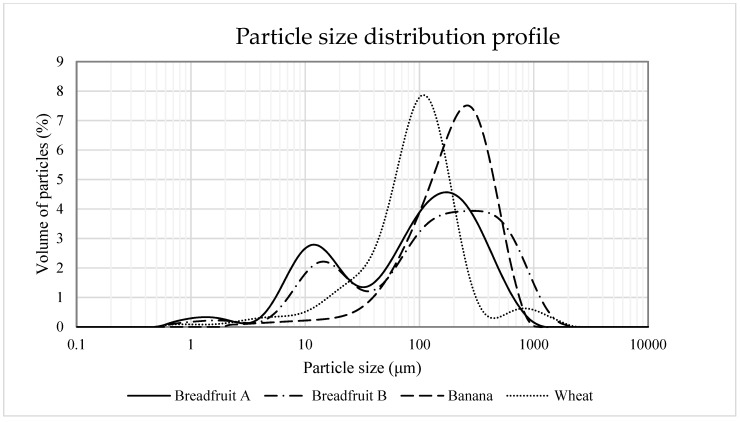
Particle size distribution profiles of tropical flours compared with a traditional flour. Treatments were flour types of breadfruit A, breadfruit B, banana, or wheat. Breadfruit flour A was sourced from Natural Foods International (Apia, Western Samoa). Breadfruit flour B was sourced from Jungle Project (Alajuela, Costa Rica). Banana flour was sourced from LiveKuna, Kunachia LLC (Davie, FL, USA). Wheat flour was obtained commercially from Bulk Barn Foods (Aurora, ON, Canada).

Water holding capacity is desirable in most food processing systems to improve yield and provide the appropriate organoleptic properties that make foods unique and acceptable to consumers [28]. Oil holding capacity is also an important physical property for food products since lipids often improve flavor and texture of foods [29]. Breadfruit flour B had the greatest (*p* < 0.05) water holding capacity, followed by banana flour and breadfruit flour A, while wheat flour had the least (*p* < 0.05) water holding capacity. Similarly, breadfruit flour B had the greatest (*p* < 0.05) oil holding capacity, followed by breadfruit flour A and wheat flour, while banana flour had the least (*p* < 0.05) oil holding capacity. Overall, the water and oil holding capacity of wheat flour was less (*p* < 0.05) compared with the breadfruit flours. Water and oil holding capacity are based on several factors such as: flour carbohydrate contents, particle size, amount of damages starch, the ratio of amylose to amylopectin in starch, and intra and inter molecular forces [30], which warrants further investigation for the purposes of this study. Particle size distribution profile was plotted (Figure 1) and the volume weighted mean (D_4,3_) which indicated the central point of the volume distribution of the particles was presented. The particle size of breadfruit flour B (220.94 μm) and banana flour (222.89 μm) were similar to those reported by Roman et al. [13]. These values were greater (*p* < 0.05) than that of breadfruit flour A (138.73 μm) and wheat flour (132.54 μm). As depicted in the plotted particle size distribution profile, bimodal distributions were observed for breadfruit flour A and breadfruit flour B, which likely indicated the distinct separation of particle size between starch granules and flour particles with these flours. Several previous research studies have described that differences in processing techniques affected particle size distribution, which may have meaningful effects on a variety of chemical and physical properties [31,32,33]. The results in this study also indicated that the flours with greater particle size and/or greater carbohydrate content might have greater water holding capacity. 

Bulk density is important for determining packaging and material handling requirements in the food industry. As an example, this includes the design of metering equipment such as particulate solid feeders. Bulk density is generally affected by flour density [34]. Greater bulk density is a desirable physical property for the following reasons: 1) a greater quantity can be packed within a constant volume, and thus offering greater packaging advantage [35]; and 2) particles are flow with more freedom which improves processing flexibility. Breadfruit flour B had the lowest (*p* < 0.05) bulk density, while banana flour had the greatest (*p* < 0.05) bulk density.

#### 3.2.2. Starch Gelatinization

**Figure 2:**
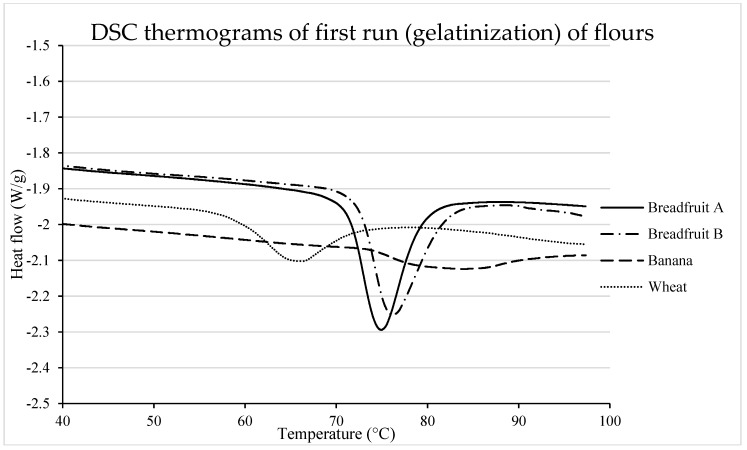
DSC thermograms (gelatinization) of tropical flours compared with a traditional flour. Treatments were flour types of breadfruit A, breadfruit B, banana, or wheat. Breadfruit flour A was sourced from Natural Foods International (Apia, Western Samoa). Breadfruit flour B was sourced from Jungle Project (Alajuela, Costa Rica). Banana flour was sourced from LiveKuna, Kunachia LLC (Davie, FL, USA). Wheat flour was obtained commercially from Bulk Barn Foods (Aurora, ON, Canada).

The melting of double helices of starch chains was evaluated through DSC (Table 3, Figure 2). Flours from tropical fruits (especially banana flour) were characterized with greater (*p* < 0.05) T_0_, T_p_, and T_c_ when compared with wheat flour. This could be the result of greater heat stability of double helices comprised by longer amylopectin chains. In fact, Roman et al. reported greater gelatinization temperatures for banana starch, which was attributed to the longer population of short chains (i.e., A and B1 amylopectin chains) [13]. These results would suggest that breadfruit starch contains amylopectin with a longer population of short chains than those from wheat starch, although shorter than those reported for banana starch. Meanwhile, the enthalpy of the gelatinization peak was in the order of breadfruit flour, wheat flour, and then banana flour, which is assumed to be related to the amylose: amylopectin ratio. Therefore, breadfruit flours are assumed to have lower amylose: amylopectin ratio than wheat, whereas banana starch would have a high amylose ratio, which would agree with results previously reported by Roman et al. [13].

#### 3.2.3. Solubility and Swelling Power

**Figure 3:**
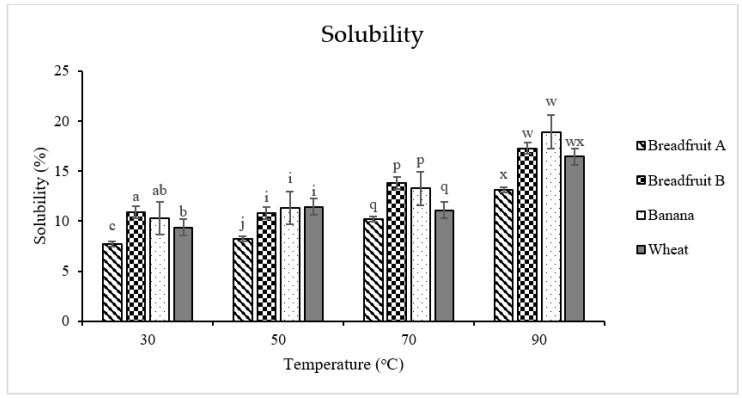
Solubility of tropical flours compared with a traditional flour. Within each temperature, means without a common superscript differ (*p* ≤ 0.05). Treatments were flour types of breadfruit A, breadfruit B, banana, or wheat. Breadfruit flour A was sourced from Natural Foods International (Apia, Western Samoa). Breadfruit flour B was sourced from Jungle Project (Alajuela, Costa Rica). Banana flour was sourced from LiveKuna, Kunachia LLC (Davie, FL, USA). Wheat flour was obtained commercially from Bulk Barn Foods (Aurora, ON, Canada).

Solubility is determined as the percentage of dissolved flour from a heated solution. The solubility generally increased when the temperature increased from 30 °C to 90 °C for each type of flour (Figure 3). Breadfruit flour A was least (*p* < 0.05) soluble at all the evaluated temperatures (from 30 °C to 90 °C) when compared with the other flour sources (with the exceptions of no significant difference at 70 °C and 90 °C between the breadfruit flour A and wheat flour). Solubilized amylose molecules leach from swelled starch granules [36]. Lower solubility may be due to the lower amylose content, which together with DSC results, would suggest that the starch contained in breadfruit flours possesses a low amylose ratio.

**Figure 4:**
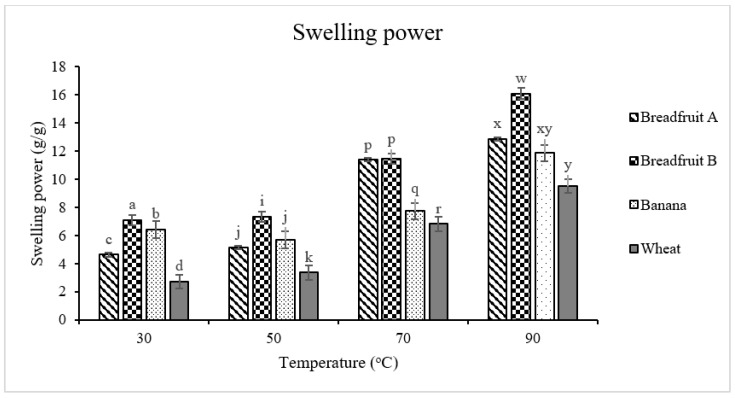
Swelling power of tropical flours compared with a traditional flour. Within each temperature, means without a common superscript differ (*p* ≤ 0.05). Treatments were flour types of breadfruit A, breadfruit B, banana, or wheat. Breadfruit flour A was sourced from Natural Foods International (Apia, Western Samoa). Breadfruit flour B was sourced from Jungle Project (Alajuela, Costa Rica). Banana flour was sourced from LiveKuna, Kunachia LLC (Davie, FL, USA). Wheat flour was obtained commercially from Bulk Barn Foods (Aurora, ON, Canada).

Swelling power in reference to flours is the ability of the flour to imbibe water when heated in an aqueous suspension and is defined as the swollen sediment weight per g of dry flour. Generally, for each type of flour, the swelling power increased when the temperature increased from 30 °C to 90 °C (Figure 4). Swelling power of each flour was more pronounced from 70 °C and onto 90 °C, and this might be because the cooking temperature was close to the starch gelatinization of the flours. The swelling power of the wheat flour was lower (*p* < 0.05) than the tropical flours at from 30 °C to 70 °C. At 70 °C, two breadfruit flours had greater (*p* < 0.05) swelling power compared with banana flour and wheat flour indicating the overall weaker granule structure of the former flour types. Swelling power is a measure of hydration capacity and when flour is heated to above the gelatinization range in excess water, hydrogen bonds stabilizing the structure of the double helices in crystallites are disrupted and are embraced with water, thus leading to flour swelling and increased overall volume [37].

#### 3.2.4. Re-association of Starch Molecules During Retrogradation

**Figure 5:**
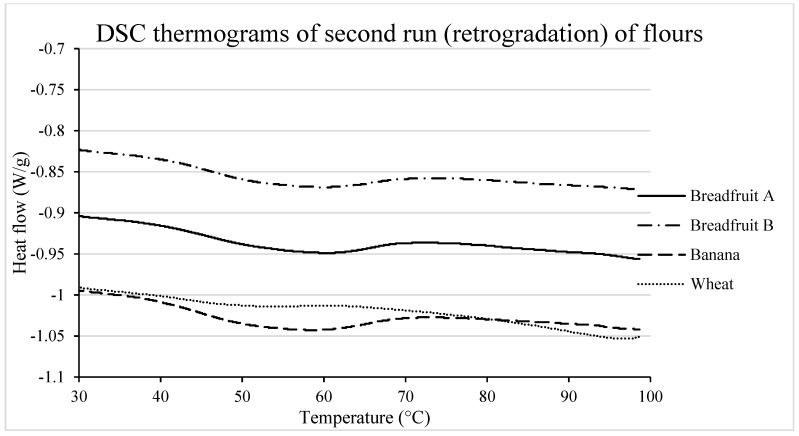
DSC thermograms (retrogradation) of tropical flours compared with a traditional flour. Treatments were flour types of breadfruit A, breadfruit B, banana, or wheat. Breadfruit flour A was sourced from Natural Foods International (Apia, Western Samoa). Breadfruit flour B was sourced from Jungle Project (Alajuela, Costa Rica). Banana flour was sourced from LiveKuna, Kunachia LLC (Davie, FL, USA). Wheat flour was obtained commercially from Bulk Barn Foods (Aurora, ON, Canada).

During storage, amylopectin chains tend to re-associate with other amylose and/or amylopectin chains through the formation of double helices or aggregates of double helices [38]. The thermal transitions and enthalpy of this endothermal peak in samples stored for 7 days, which corresponds to retrograded AP, are summarized in Table 3 and Figure 5. Results showed greater peak (T_p_) and conclusion (T_c_) temperatures for the melting of retrograded amylopectin in tropical fruits compared to that from wheat, indicating that the amylopectin in tropical fruits is formed by longer A and B1 chains [39]. The wider temperature range would in turn indicate a greater amount of heterogeneous crystals [40]. Likewise, tropical fruit flours, especially that from banana, exhibited a dramatically greater retrogradation enthalpy compared with wheat flour, indicating a high propensity of tropical fruit flour amylopectin re-associated during storage. It is noteworthy that starch retrogradation would contribute to high measured enthalpy of retrogradation (ΔHr) for the flours, but not necessarily to their influence to affect negatively texture during storage [41].

#### 3.2.5. Pasting Properties

**Figure 6:**
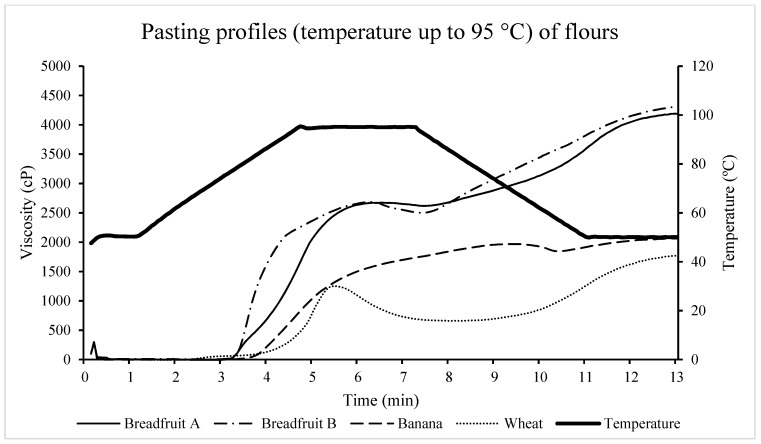
The pasting properties of tropical flours compared with a traditional flour using RVA method #1 (maximum temperature of 95 °C). Treatments were flour types of breadfruit A, breadfruit B, banana, or wheat. Breadfruit flour A was sourced from Natural Foods International (Apia, Western Samoa). Breadfruit flour B was sourced from Jungle Project (Alajuela, Costa Rica). Banana flour was sourced from LiveKuna, Kunachia LLC (Davie, FL, USA). Wheat flour was obtained commercially from Bulk Barn Foods (Aurora, ON, Canada).

The absence of a well-developed peak was found in the pasting results of the first RVA method (temperature was increased up to 95 °C) (Table 4, Figure 6), which indicates that starch granules did not fully swell. Moreover, the banana flour seems to be the most resistant to heating and swelling at 95 °C. The pasting results of the first RVA method were similar to the results of other research studies [36,42,43], where the test temperature was also up to 95 °C.

**Figure 7:**
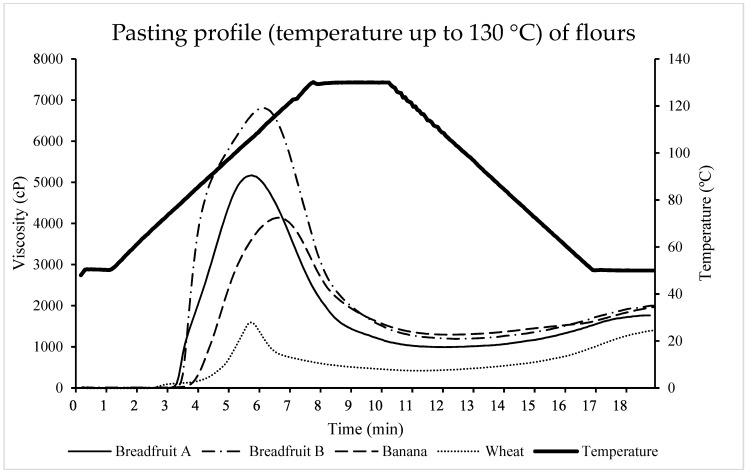
The pasting properties of tropical flours compared with a traditional flour using RVA method #2 (maximum temperature of 130°C). Treatments were flour types of breadfruit A, breadfruit B, banana, or wheat. Breadfruit flour A was sourced from Natural Foods International (Apia, Western Samoa). Breadfruit flour B was sourced from Jungle Project (Alajuela, Costa Rica). Banana flour was sourced from LiveKuna, Kunachia LLC (Davie, FL, USA). Wheat flour was obtained commercially from Bulk Barn Foods (Aurora, ON, Canada).

For the pasting results of the second method (temperature was increased to 130 °C), the peak viscosity, trough viscosity, and final viscosity of the tropical flours, was greater (*p* < 0.05) compared with wheat flour (Table 5). The differences of viscosity might be explained by the combination effects of the test conditions, the composition of carbohydrates in the flour, the ratio of amylose to amylopectin, and the resistance of their starch granules to swell. The peak time of tropical flours was longer (*p* < 0.05) than that of the wheat flour. However, pasting temperature did not statistically differ (*p* = 0.46) among treatments. Moreover, the viscosity of all the flours decreased during temperature holding at 130 °C (Figure 7). The breakdown of the tropical flours was greater (*p* < 0.05) than wheat flour. The peak viscosity and the breakdown were much greater than those of using the first RVA method. The differences between using the first and the second RVA method may be caused by different components such as protein and fat were attached to starch granules, which leads to uncompleted gelatinization process of the flours under 100 °C. The flours might need higher temperature to become fully gelatinized. It is interesting to note that the tropical flours had extraordinary swelling power, especially breadfruit flour, although they did not resist heating and the viscosity decreased significantly.

## 4. Conclusions

The tropical fruit flours (breadfruit flours and the banana flour) had greater water holding capacity and oil holding capacity compared with the traditional flour source (wheat flour). A high propensity of tropical fruit flour amylopectin re-associated during storage since tropical fruit flours exhibited a dramatically greater retrogradation enthalpy compared with wheat flour. The tropical flours had greater the peak viscosity, trough viscosity, and final viscosity compared to wheat flour. Moreover, the tropical flours did not resist heating but had extraordinary swelling power. Based on the results of flour composition, functional properties, and pasting properties, it is reasonable to conclude that both of these sources of flour (breadfruit flour and banana flour) may serve as useful ingredients and substitute the traditional flour such as, wheat flour in food products (e.g., breads, pancakes, noodles, and meat products) with improved functional properties (e.g., hydration, emulsification, fat retention, coagulation, heat stability, etc.). The next stage of this research would be to investigate the use of these sources of flour in several different food applications.

## Figures and Tables

**Table 1:** Proximate composition of tropical flours compared with a traditional flour.

Items	Units	Breadfruit A ^1^	Breadfruit B ^2^	Banana ^3^	Wheat ^4^	RDL ^5^
***As is basis***						
Moisture	g/100 g	12.60	6.47	8.18	12.20	0.1
Carbohydrates	g/100 g	80.13	83.98	83.24	72.45	0.1
Starch	g/100 g	64.14	62.68	70.83	60.40	0.1
Protein	g/100 g	3.42	4.67	3.98	12.74	0.10
Fat	g/100 g	0.85	2.34	1.78	1.51	0.10
Ash	g/100 g	3.00	2.54	2.82	1.10	0.1
Energy	kJ/100 g	1430.30	1571.81	1526.70	1482.60	1.0
Calories	kcal/100 g	341.85	375.67	364.89	354.35	1.0
***Dry matter basis***						
Carbohydrates	g/100 g	91.68	89.79	90.66	82.52	
Starch	g/100 g	73.39	67.02	77.14	68.79	
Protein	g/100 g	3.91	4.99	4.33	14.51	
Fat	g/100 g	0.97	2.50	1.94	1.72	
Ash	g/100 g	3.43	2.72	3.07	1.25	
Energy	kJ/100 g	1636.50	1680.54	1662.71	1688.61	
Calories	kcal/100 g	391.13	401.66	397.40	403.59	

^1^ Breadfruit flour A was sourced from Natural Foods International (Apia, Western Samoa); ^2^ Breadfruit flour B was sourced from Jungle Project (Alajuela, Costa Rica); ^3^ Banana flour was sourced from LiveKuna, Kunachia LLC (Davie, FL, USA); ^4^ Wheat flour was obtained commercially from Bulk Barn Foods (Aurora, ON, Canada); ^5^ RDL = reportable detection limit.

**Table 2:** The functional properties of tropical flours compared with a traditional flour ^1^.

	Breadfruit A ^2^	Breadfruit B ^3^	Banana ^4^	Wheat ^5^	SEM	*p*-Value
pH	6.09 ^a^	5.67 ^c^	5.07 ^d^	5.84 ^b^	0.02	<0.0001
WHC ^6^, g/g	2.04 ^c^	3.32 ^a^	3.08 ^b^	1.25 ^d^	0.02	<0.0001
OHC ^7^, g/g	1.71 ^b^	2.15 ^a^	0.94 ^d^	1.10 ^c^	0.04	<0.0001
BD ^8^, g/cm^3^	0.70 ^c^	0.52 ^d^	0.94 ^a^	0.78 ^b^	0.01	<0.0001
D_4,3_^9^, um	138.73 ^b^	220.94 ^a^	222.89 ^a^	132.54 ^b^	10.75	<0.001

^a–d^ Least square means within a row with different superscripts are statistically different (*p* < 0.05); ^1^ Data presented are LS means and reported SEM is the maximum standard error of the mean among treatments; ^2^ Breadfruit flour A was sourced from Natural Foods International (Apia, Western Samoa); ^3^ Breadfruit flour B was sourced from Jungle Project (Alajuela, Costa Rica); ^4^ Banana flour was sourced from LiveKuna, Kunachia LLC (Davie, FL, USA); ^5^ Wheat flour was obtained commercially from Bulk Barn Foods (Aurora, ON, Canada); ^6^ Water holding capacity, expressed as the weight (in g) of water bound by 1 g of dried flour sample; ^7^ Oil holding capacity, expressed as the weight (in g) of oil bound by 1 g of dried flour sample; ^8^ Bulk density; ^9^ D_4,3_ Volume weighted mean indicates the central point of the volume distribution of the particles.

**Table 3:** Gelatinization and retrogradation properties of tropical flours compared with a traditional flour ^1^.

	Breadfruit A ^2^	Breadfruit B ^3^	Banana ^4^	Wheat ^5^	SEM	*p*-Value
***Gelatinization***						
T_o_ (°C)	71.33 ^c^	72.45 ^b^	74.19 ^a^	59.21 ^d^	0.23	<0.0001
T_p_ (°C)	75.06 ^c^	75.97 ^b^	82.94 ^a^	65.47 ^d^	0.14	<0.0001
T_c_ (°C)	88.03 ^b^	88.39 ^b^	96.20 ^a^	77.49 ^c^	0.26	<0.0001
T_c_–T_o_ (°C)	16.70 ^bc^	15.94 ^c^	22.01 ^a^	18.28 ^b^	0.45	<0.0001
ΔH (W/g)	12.98 ^a^	12.31 ^a^	3.56 ^c^	6.30 ^b^	0.25	<0.0001
***Retrogradation***						
T_o_ (°C)	40.67	40.73	38.97	42.69	0.99	0.15
T_p_ (°C)	57.77 ^a^	55.66 ^a^	55.51 ^a^	51.77 ^b^	0.50	<0.001
T_c_ (°C)	72.96 ^b^	73.70 ^ab^	75.32 ^a^	60.48 ^c^	0.52	<0.0001
T_c_–T_o_ (°C)	32.29 ^a^	32.97 ^a^	36.35 ^a^	17.79 ^c^	0.95	<0.0001
ΔH (W/g)	5.50 ^b^	5.74 ^b^	6.99 ^a^	0.74 ^c^	0.14	<0.0001

^a–d^ Least square means within row with different superscripts are statistically different (*p* < 0.05); ^1^ Data presented are LS means and reported SEM is the maximum SEM among treatments. T_o_-onset temperature, T_p_-peak temperature, T_c_-conclusion temperature, ΔH- enthalpy; ^2^ Breadfruit A was sourced from Natural Foods International (Apia, Western Samoa); ^3^ Breadfruit B was sourced from Jungle Project (Alajuela, Costa Rica); ^4^ Banana flour was sourced from (Kunachia LLC, Davie, FL, USA); ^5^ Wheat flour was obtained commercially from Bulk Barn Foods (Aurora, ON, Canada)

**Table 4:** The pasting properties of tropical flours compared with a traditional flour using RVA method #1 (maximum temperature of 95 °C) ^1^.

	Breadfruit A ^2^	Breadfruit B ^3^	Banana ^4^	Wheat ^5^	SEM	*p*-Value
Peak viscosity ^6^, cP	2678.33 ^a^	2687.33 ^a^	1701.00 ^b^	1258.67 ^c^	34.85	<0.0001
Trough Viscosity ^7^, cP	2613.33 ^a^	2499.33 ^a^	1524.33 ^b^	658.00 ^c^	36.28	<0.0001
Breakdown ^8^, cP	65.00 ^c^	188.00 ^b^	176.67 ^b^	600.67 ^a^	5.42	<0.0001
Final Viscosity ^9^, cP	4188.67 ^a^	4310.00 ^a^	2063.33 ^b^	1768.00 ^c^	39.31	<0.0001
Setback ^10^, cP	1575.33 ^b^	1810.67 ^a^	539.00 ^d^	1110.00 ^c^	22.53	<0.0001
Peak time ^11^, min	6.60 ^b^	6.36 ^b^	7.00 ^a^	5.44 ^c^	0.08	<0.0001
Pasting temperature ^12^, °C	76.80 ^c^	76.75 ^c^	82.40 ^b^	86.85 ^a^	0.14	<0.0001

^a–d^ Least square means within a row with different superscripts are statistically different (*p* < 0.05); ^1^ Data presented are LS means and reported SEM is the maximum SEM among treatments; ^2^ Breadfruit flour A was sourced from Natural Foods International (Apia, Western Samoa); ^3^ Breadfruit flour B was sourced from Jungle Project (Alajuela, Costa Rica); ^4^ Banana flour was sourced from LiveKuna, Kunachia LLC (Davie, FL, USA); ^5^ Wheat flour was obtained commercially from Bulk Barn Foods (Aurora, ON, Canada); ^6^ Peak viscosity indicates the water holding capacity of the flour and the viscous load likely to be encountered by a mixing cooker.; ^7^ Tough viscosity is the minimum viscosity after peak; ^8^ Breakdown = peak viscosity − trough viscosity; ^9^ Final viscosity is the viscosity at the end of the test, which indicates the ability of the flour to form a viscous paste or gel after cooking and cooling; ^10^ Setback = final viscosity − trough viscosity; ^11^ Peak time is the time when peak viscosity occurred; ^12^ Pasting temperature indicates the minimum temperature required to cook the flour sample and indicates energy costs.

**Table 5:** The pasting properties of tropical flours compared with a traditional flour using RVA method #2 (maximum temperature of 130 °C) ^1^.

	Breadfruit A ^2^	Breadfruit B ^3^	Banana ^4^	Wheat ^5^	SEM	*p*-Value
Peak viscosity ^6^, cP	5169.33 ^b^	6809.00 ^a^	4142.00 ^c^	1597.00 ^d^	76.95	<0.0001
Trough Viscosity ^7^, cP	991.67 ^c^	1195.33 ^b^	1296.67 ^a^	418.33 ^d^	12.43	<0.0001
Breakdown ^8^, cP	4177.67 ^b^	5613.67 ^a^	2845.33 ^c^	1178.67 ^d^	67.72	<0.0001
Final Viscosity ^9^, cP	1766.00 ^b^	2000.33 ^a^	1962.00 ^a^	1396.00 ^c^	42.03	0.0001
Setback ^10^, cP	774.33 ^b^	805.00 ^ab^	665.33 ^b^	977.67 ^a^	37.30	<0.01
Peak time ^11^, min	5.85 ^c^	6.11 ^b^	6.58 ^a^	5.71 ^d^	0.02	<0.0001
Pasting temperature ^12^, °C	77.85	78.45	82.73	76.53	2.71	0.46

^a–d^ Least square means within a row with different superscripts are statistically different (*p* < 0.05); ^1^ Data presented are LS means and reported SEM is the maximum SEM among treatments; ^2^ Breadfruit A was sourced from Natural Foods International (Apia, Western Samoa); ^3^ Breadfruit B was sourced from Jungle Project (Alajuela, Costa Rica); ^4^ Banana flour was sourced from LiveKuna, Kunachia LLC (Davie, FL, USA); ^5^ Wheat flour was obtained commercially from Bulk Barn Foods (Aurora, ON, Canada); ^6^ Peak viscosity indicates the water holding capacity of the flour and the viscous load likely to be encountered by a mixing cooker; ^7^ Tough viscosity is the minimum viscosity after peak; ^8^ Breakdown = peak viscosity − trough viscosity; ^9^ Final viscosity is the viscosity at the end of the test, which indicates the ability of the flour to form a viscous paste or gel after cooking and cooling; ^10^ Setback = final viscosity − trough viscosity; ^11^ Peak time is the time when peak viscosity occurred; ^12^ Pasting temperature indicates the minimum temperature required to cook the flour sample and indicates energy costs.
